# Domestic abuse experienced by healthcare practitioners: what about dentists and dental care professionals? A systematised review

**DOI:** 10.1038/s41415-025-9011-x

**Published:** 2025-12-12

**Authors:** Lorna K. MacNab, Paul Cannon, Clare McFeely, Sandi Dheensa

**Affiliations:** 121935361515442804909 Consultant and Honorary Clinical Senior Lecturer in Restorative Dentistry, Glasgow Dental Hospital and School, United Kingdom; 835059233698589946800 University Library, University of Glasgow, United Kingdom; 987352569850821560645 Senior Lecturer, Medicine, Dentistry and Nursing, University of Glasgow, United Kingdom; 555163550270803134920 Research Fellow, Bristol Medical School, United Kingdom

## Abstract

**Introduction** Domestic abuse (DA) is a significant global public health issue. It is recognised that dentists and dental care professionals (DCPs) play a key role in recognising and responding to abuse in patients; however, it is rarely acknowledged that they, personally, may be DA victim/survivors.

**Method and results** A systematised review of the literature found no specific reference to dentists or DCPs as DA victims/survivors. This systematised review focused exclusively on dentists and DCPs and found no papers on the topic of dentists'/DCPs' own lived experiences of DA. In the absence of any evidence specific to this healthcare professional (HCP) group, evidence drawn from other healthcare groups (e.g., nurses, midwives, doctors) is considered as a basis for informing likely impacts of DA on dentists, DCPs and the dental workplace. Dental staff victims/survivors are likely to encounter similar additional barriers to help-seeking as other HCPs. These will be specific to their working role and environment.

**Discussion and conclusion** Employers have a statutory duty to ensure that the working environment is safe and supportive, and this includes considering the impact of DA on their employees. The workplace can play a vital role in supporting dental staff who are DA victims/survivors. Research is needed to understand the prevalence and nature of the lived experience of DA among dentists and DCPs and to address issues specific to dental working environments. DA among staff must be acknowledged by employers of dentists and DCPs both in the NHS and in private healthcare settings to ensure suitable policies, education and training are in place.

**Supplementary Information:**

Zusatzmaterial online: Zu diesem Beitrag sind unter 10.1038/s41415-025-9011-x für autorisierte Leser zusätzliche Dateien abrufbar.

## Key points

- Domestic abuse is a global public health issue with significant personal and societal consequences.
- There is an expectation that dentists and dental care professionals should recognise and respond to patients affected by domestic abuse but little recognition that they may be victims/survivors themselves.
- Dentists and dental care professionals may experience additional barriers to disclosure of personally experienced abuse.
- The workplace may be the only source of respite so increased awareness and targeted support methods are essential.

**Supplementary Information:**

Zusatzmaterial online: Zu diesem Beitrag sind unter 10.1038/s41415-025-9011-x für autorisierte Leser zusätzliche Dateien abrufbar.

## Introduction

### Definition and prevalence

Domestic abuse (DA) is a pervasive global public health issue adversely affecting individuals, families, communities and the workplace and is a violation of an individual's human rights. Abusers aim to exert power and control over victims/survivors to create dependency or subordination, via a pattern of controlling, coercive, threatening and degrading behaviour, often including physical and sexual violence (Box 1).^1^^,^^2^ It is never the victims/survivor's fault. DA is defined in Scotland as perpetrated by a partner/ex-partner, and in England, Wales and Northern Ireland by (ex)partners or relatives.^3^^,^^4^^,^^5^^,^^6^ DA is common: in England and Wales, 6.6% of women (1.6 million) and 3.0% of men (712,000) aged 16 years or over reported past-year experience.^7^

Box 1 Domestic abuse examples of controlling and coercive behaviours^2^

- Making someone dependent on or subordinate to the abuser
- Isolating someone from their friends, relatives, or other sources of support
- Controlling, regulating, or monitoring someone's day to day activities
- Depriving someone of, or restricting their freedom of, action, e.g., controlling their phone/communication access or access to money
- Frightening, humiliating, degrading or punishing someone, e.g., abusive name calling, playing mind games that causes someone to doubt their sanity.

### Impact of domestic abuse

DA has long-lasting physical and mental health consequences, including direct injury, even post-separation, and may be fatal.^8^ DA can affect anyone, but disproportionately affects women,^1^^,^^8^^,^^9^ with 72.5% of DA-related crimes having a female victim.^7^ DA is a cause and consequence of gender inequality.^10^ Women are more likely to experience repeat victimisation, physical injury or homicide, and sexual violence.^9^ Intersecting forms of oppression and discrimination may create additional barriers to disclosure and help-seeking.^11^

### Impact in the workplace

DA can extend to the professional environment. Research shows 75% of survivors are targeted at work, e.g., harassment and stalking.^12^ DA can include sabotaging transport to work and threats and intimidation to colleagues.^13^ Colleagues may need to cover workload while survivors take leave to recover from abuse or attend court. When the survivor and abuser are colleagues, survivors may face risk and be fearful during and after relationships; this dynamic may be difficult for colleagues to manage if DA has been divulged. Perpetrators may go undetected in the workplace as they can appear professional. Thus, there may be an impact on attendance, confidence, performance and professional relationships. This can result in financial consequences for the individual and the wider economy. DA cost the economy an estimated £66 billion in 2016–17: £14 bn from lost output.^14^

### National responses

In England, DA cases are among the highest priority work dealt with in the criminal justice system.^15^ Recently, NHS Scotland (National Health Service) has invested in enhancing the healthcare professionals' (HCPs) role in recognising and responding to people affected, while a National DA Commissioner has been appointed in England and Wales and a DA Lead appointed for NHS England.

### Healthcare professionals as victims/survivors

DA prevalence may be higher among HCPs.^16^^,^^17^ Nurses, midwives and healthcare support workers report rates of past-year DA three times higher than the general population (14% versus 4.4%).^17^ In 2022, Dheensa and colleagues' systematic review and meta-analysis of 48 studies across 19 countries found a lifetime experience of 41.8% for female HCPs and 14.8% for male HCPs.^18^ Most staff were doctors, nurses, midwives and mental health staff.^18^

### What about dentists and dental care professionals?

Dentists and dental care professionals (DCPs) were not represented in any of the studies; however, Dheensa *et al.*'s findings on essential challenges should be applicable to these groups. HCPs, including dentists and DCPs, have a key role identifying, responding to and referring those affected, as well as treating physical injuries,^19^^,^^20^^,^^21^^,^^22^ which are mostly to the head and neck.^21^^,^^23^^,^^24^ Dentists and dental teams, therefore, are expected to recognise signs of abuse in acute presentations following assault, in routine consultation or as a primary source of associated dental issues.^21^^,^^23^^,^^24^ However the possibility that the dentist or DCP themselves might have experienced DA is under-acknowledged.

All the DA impacts described above are equally applicable to dentists and DCPs; however, Dheensa's reviewed papers did not consider dentists' or DCPs' experiences specifically, nor mentioned them in the text. The review's search strategy did not include dental terms, and the grey literature search did not specify the British Dental Association (BDA) or the General Dental Council (GDC). Dentistry DA research focuses on the clinician's role of providing care for survivors and may, unintentionally, reinforce the stereotype of dentist and DCP in the role of healthcare worker and not perceivable as a victim/survivor. Given the significant personal risks to individual dentists and DCPs, the impact on the workplace and wider economy, and the potential for employers to provide support and safety, it is essential that the subject of dentists' and DCPs' own lived experience is researched.

## Aims, objectives and method

We conducted a systematised literature review to explore dentists' and DCPs' own, lived experience of DA. Systematised reviews employ a transparent, systematic approach to searching, screening and synthesising literature.^25^

### Inclusion criteria

Lived experience of DA in dentists and DCPs (dental nurses, dental technicians, dental therapists, dental hygienists, orthodontic therapists, clinical dental technicians).

### Exclusion criteria

Articles about: gender-based violence not specific to DA; children's experience (although DA is closely associated with child protection issues); undergraduate dental students (to align with Dheensa *et al.*'s review). We adopted ethical guidance for DA research to report our findings.^26^

### Information sources

Building on Dheensa and colleagues' search terms (2022), PC developed a comprehensive search encompassing subject headings and free-text terms for DA and dentistry.^18^ The search was limited to the United Kingdom (UK) in order to inform UK workplace action using the validated filter developed by Ayiku *et al.* (2019).^27^ LM and CM reviewed and augmented a pilot Medline search, then translated it for three (Medline, Cinhal, Embase) from inception to 21 August 2023. Searches were limited to English language, with no additional restrictions (see online Supplementary Information for details).

## Results

**Fig. 1:**
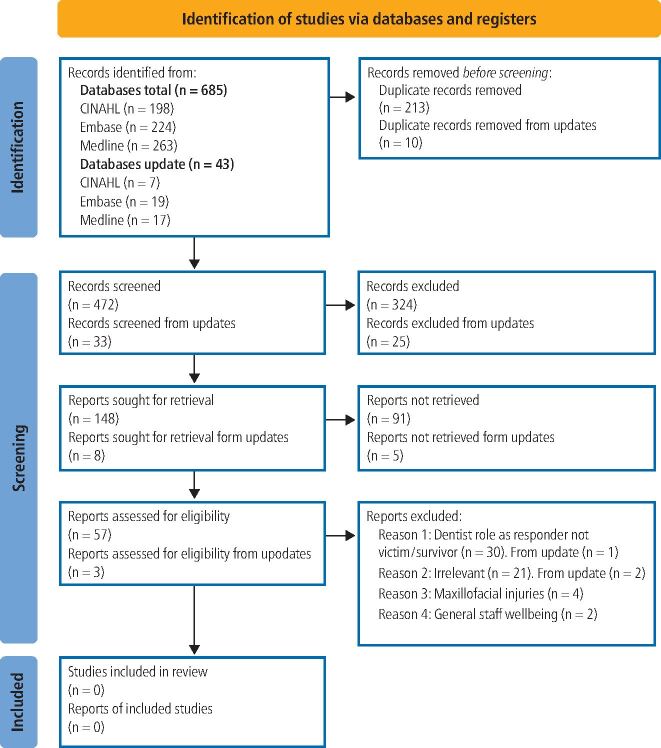
PRISMA flow diagram

LM screened 472 potentially relevant studies by title, 148 abstracts, and 57 full articles (Fig. 1).^28^ Most explored the dentist's role responding to victims/survivors and managing DA-related dental-maxillofacial injuries. One article discussed dentists' wellbeing, non-specific to DA. No papers about dentists'/DCPs' lived experiences of DA were found. No reference to dentists as victim/survivors of abuse were found. This identifies an important gap in the evidence base.

## Discussion

The lack of published papers describing dentists' and DCPs' own experience of DA demonstrates that the existence, importance and impact of this issue has not been recognised. The extent of the issue is, therefore, unknown. In the absence of any data specific to dentists and DCPs, it is reasonable to assume that papers relating to DA experienced by other HCPs are likely to be applicable to dentists and DCPs.^16^^,^^17^^,^^18^

### Impact in the healthcare workplace

The healthcare workplace impact is described in Dheensa and colleagues' review. Victims/survivors will, at times, be less able to work to their best ability (e.g., reduced concentration/feeling too unwell to work). Those who continue to work may experience reduced confidence or worry about making a mistake, compounding stress. Employers may misinterpret behaviours such as lateness and instigate disciplinary action.^18^^,^^29^ Victims/survivors were more able to identify and respond to DA among patients but doing so could be distressing.^18^^,^^29^

### Barriers to disclosure for all victims/survivors

While a range of agencies can offer support (Box 2), all victims/survivors can find disclosure and help-seeking difficult.^30^ All victims/survivors may be aware of abuse and reluctant to disclose, or may not recognise abuse, often because the cycle of abuse has grown and been normalised over a long period.^30^ Other barriers to disclosure include fear of perpetrator retaliation, the potential impact on and fear of removal of children, isolation, shame, discouragement from close relatives, victim-blaming societal attitudes, fearing pressure to exit the relationship immediately, and lacking practical and financial resources.^31^^,^^32^ Victims are at highest risk when reporting or leaving the abuser.

Box 2 Where to get support

- In an emergency, call 999
- Talk to a doctor, health visitor or midwife
- Women can call The Freephone National Domestic Abuse Helpline, run by Refuge on 0808 2000 247 for free at any time, day or night. Online chat is also available
- Women can email helpline@womensaid.org.uk. Staff will respond to your email within five working days or search the Domestic Abuse Directory at https://www.womensaid.org.uk/womens-aid-directory/ to find local support
- Men can call Respect Men's Advice Line on 0808 8010 327 (Monday to Friday 10 am to 8 pm) or visit the webchat at Men's Advice Line (Wednesday 10 am to 11.30 am and 2.30 pm to 4 pm) for non-judgemental information and support info@mensadviceline.org.uk
- Men can also call ManKind on 0182 3334 244 (Monday to Friday, 10 am to 4 pm) or Freephone 08088001170
- National LGBT+ Domestic Abuse Helpline (run by Galop) on 0800 999 5428 for emotional and practical support or email help@galop.org.uk
- The Sistah Space is a specialist charity that supports African and Caribbean heritage women affected by domestic and sexual abuse, UK-wide
- Anyone can call Karma Nirvana on 0800 5999 247 (Monday to Friday 9 am to 5 pm) for forced marriage and honour-based abuse. You can also call 020 7008 0151 to speak to the GOV.UK Forced Marriage Unit
- The Mix offers free information and support for under 25s in the UK 0808 808 4994
- If you are worried that you are abusive, you can contact the free Respect helpline on 0808 802 4040 or via the webchat service on the charity's website
- Scotland's 24-hour Domestic Abuse and Forced Marriage Helpline on 0800 027 1234 or visit sdafmh.org.uk
- Women's Aid Federation Northern Ireland https://www.womensaidni.org/get-help/ Domestic and Sexual Abuse Helpline 0808 802 1414

**Additional support:**

- ASSIST: Independent Domestic abuse Advocacy for Court in Scotland https://www.yoursupportglasgow.org/directory/providerdetails/52089 0141 276 7710
- Rights of Women (rightsofwomen.org.uk)
- Scottish Women's Rights Centre (civil matters only) https://www.scottishwomensrightscentre.org.uk
- Victim Support England and Wales 0808 1689 111
- Victim Information and Advice https://www.copfs.gov.uk/services/victim-services/victim-information-and-advice-via-service/
- The Survivor's Handbook from the charity Women's Aid is free and provides information for women on a wide range of issues, such as housing, money, helping your children, and your legal rights.

### Additional barriers for healthcare professionals

HCPs appear to experience additional barriers to disclosure, as outlined below.^18^^,^^33^^,^^34^^,^^35^^,^^36^All barriers have the effect of silencing victims/survivors and sustaining risk.

#### Stereotypes, stigma and shame

The contrast between stereotypes of successful, autonomous professionals and ‘abused people' reduces the likelihood of self-awareness, disclosure, and enquiry by an HCP, and adds to fears of disbelief if they disclose.^35^ Some female medics who attempted to disclose described negative responses, even from their own general practitioners, including closing discussions down, pressure to report abuse to police, expectations they would return to work as usual, or feelings of being a ‘nuisance', as well as fears that disclosure could raise questions about their professional capability.^35^

Donovan *et al.,* found that female medics reported an expectation that, as doctors, they are hardworking and determined to succeed, and this should apply to managing their personal life.^36^ In consequence, they described ‘going beyond limits to make things work' and the sense of failure: ‘it shouldn't happen to a doctor'. A sense of shame, compounded by societal victim-blaming may relate to expectations that doctor victims/survivors ought to ‘know better' than engage in an abusive relationship and are not supposed to be vulnerable.^35^ We posit that this resilience may, in part, increase the likelihood of remaining in an abusive relationship but this requires exploration in primary research.

#### Lack of anonymity and confidentiality and fear of professional consequences

HCP survivors are concerned about anonymity and the potential to encounter patients or be recognised from their professional role if accessing local support services.^18^^,^^35^ For some, a fear that disclosure of abuse will jeopardise their professional registration increases anxiety around confidentiality and anonymity.^18^^,^^35^ HCPs fear false allegations by the perpetrator to the regulator or to social services.

#### Isolation

Isolation from family and friends is a common characteristic of abuse, minimising opportunities for disclosure and emotional and practical support.^37^ Isolation may be unintentionally compounded as national recruitment for specialty training often requires movement to different locations around the UK.^18^^,^^35^

#### Barriers to healthcare professionals recognising and responding to domestic abuse

HCPs are expected to respond if they suspect DA, including in colleagues, but may not feel confident to act and or may not recognise the abuse due to reasons given above. This can compound the risks and barriers to personal support.^18^^,^^38^^,^^39^^,^^40^ This dual relationship between non-disclosure and non-enquiry may leave victims/survivors at risk.^40^

### Workplace action

#### Benefits of working

The workplace can be a source of stress but also of safety, respite and a supportive environment for the victim/survivor.^41^ Work can provide a sense of identity, sense of agency, social support and, potentially, escape from the perpetrator. It can provide the opportunity for financial independence.^41^ Staff disclosure and help-seeking for their own experience is a recurring unintended consequence of DA staff training, highlighting a need for support mechanisms for staff.^18^^,^^35^^,^^42^ DA survivors clearly need and want support in the workplace. Employers, therefore, need to know how to provide effective and timely support for victims/survivors.^41^

#### What healthcare professionals want

HCP victims/survivors have identified fundamental support needs in the workplace, including understanding, flexibility and confidentiality, detailed in Box 3. Research has, however, identified poor responses to DA survivors in the workplace^34^^,^^35^ and that employers should review working cultures and tackle stigmatising responses to those who disclose.

Box 3 Examples of employer responses desired by healthcare employees^17^^,^^18^^,^^35^

- Open, supportive and flexible to the needs of employees
- Improve understanding among colleagues of the staff experience of domestic violence and abuse
- Acknowledgement that they may not be able to work to the best of their ability without feeling that they might lose their jobs
- Sensitive support from qualified professionals
- Domestic violence and abuse training for staff in supportive roles is recommended (especially those in supporting roles such as occupational health or human resources) and referral pathways to domestic violence and abuse agencies
- Guarantee of confidentiality
- Support options: flexible leave and working more formalised in policy.

#### Employer's responsibility

All employers have a statutory responsibility for the health and safety of their staff, including those at risk related to DA.^43^^,^^44^^,^^45^ This includes considering the impact of DA on their employees as part of their duty of care.^46^

There have been multilevel attempts to improve workplace responses to DA, e.g., England's *Tackling Domestic Abuse Plan* (2022), with its identification of workplace support as a key pillar of support for victims/survivors, placing a clear responsibility on employers.^47^ The proactive role expected of employers is restated in the Department of Business Strategy (2020).^48^ In collaboration, the Chartered Institute of Personnel and Development, together with the Equality and Human Rights Commission, produced guidelines detailing actions that employers can take to manage and support employees experiencing DA.^49^ To further support implementation, the Employers' Initiative on Domestic Abuse is a growing network of large and small businesses and organisations supporting employers to take action on DA by raising awareness and providing further guidance.^41^ NHS England is a member. Research from Canada and the United States has also emphasised the need for legislative and policy changes, employee assistance programmes, paid DA leave, and trade union support.^50^^,^^51^ The healthcare sector should enable employers to develop interventions that can better support and increase victim/survivor resilience and support-seeking ability. The British Medical Association report recommends that all trusts and health boards have a trained, designated point of contact. Those working for professional registration bodies should receive DA training and implement policies to ensure DA disclosures will not automatically harm professional registration.^18^

#### Policies

Recommendations for a safer, supportive workplace overlap with other equalities-sensitive issues, such as flexible working policies, flexibility regarding working times/location and leave.^41^ Some safety factors, however, are DA-specific, e.g., risk assessment and safety planning, requiring clear direction for managers and colleagues. Guidance and policies are readily available to guide employers (Box 4); however, few providers report a robust, current DA policy and those that do, frequently omit emotional support and advocacy.^18^ To our knowledge, neither the GDC nor the BDA have explicit DA policies for staff (though as substantial employers are expected to have in place a range of policies and support relevant for staff and managers facing such circumstances). Power and control are at the centre of DA. People disclosing DA and seeking help want information and guidance to understand and have agency over the workplace response.^41^ If an employer has a high-quality, effective written policy, this can set out the expected procedure and workplace support available and signpost to external support.^41^ Where there is a policy, regular publicisation is critical to raise awareness of it, as is the case for other policies, such as maternity leave policy.^41^

Staff-specific policies should include right to confidentiality, provision of special leave, access to flexible hours, safety planning at work, safety considerations for employee transport to work, safety planning if employee is a ‘lone-worker', changes to pay arrangements, recognition that performance may be affected, referral to occupational health, and provision of training.^35^ Clear protocols to follow on receipt of a report of DA would be beneficial for staff.^33^

In considering positive workplace responses, it is important to recognise the contribution that survivors can make to the healthcare workplace through fulfilling their professional potential and, although challenging, through using their insight to identify and respond to DA in patients and colleagues.^18^

Box 4 Workplace policies and resources The British Medical Association

Support for Doctors Affected by Domestic Abuse

- https://www.bma.org.uk/advice-and-support/nhs-delivery-and-workforce/creating-a-healthy-workplace/domestic-abuse-in-the-health-profession-report

Employers' Initiative on Domestic Abuse (EIDA)^41^

- https://www.eida.org.uk/resources/eida-handbook

Sharon's Policy template

- https://www.eida.org.uk/resources/domestic-abuse-policy-template-and-guidance-sharons-policy

NHS Employers England

- https://www.nhsemployers.org/articles/domestic-violence-and-abuse#:~:text=The%20Tackling%20Domestic%20Abuse%20Plan,victims%20and%20perpetrators%20in%20rehabilitation

NHS Scotland Guidance

- https://www.staffgovernance.scot.nhs.uk/media/1399/gender-based-violence-employee-pin-policy.pdf

### Dental workplaces

Dentists and DCPs are employed in a variety of UK NHS and privately-owned organisations of different types and sizes. These organisations range from large teaching hospitals with many staff, multi-layered management structures and dedicated HR teams, to individual dental practices with small, close-knit teams. Flexible home-working and relocation, essential for professional development, are further complicating factors. Therefore, clarification of responsibilities and guidance for employers, as well as over-arching bodies, is important to protect and support this group. It is vital that potential sources of workplace support for dentists and DCPs are harnessed.

## Conclusion

While evidence is emerging on the extent of abuse among HCPs, and workplaces endeavour to develop support, dentists and DCPs are overlooked. Since carrying out the research for this paper, Dheensa *et al.*'s (2024) paper is the first to include dentists and DCPs in a sample of HCP DA survivors.^29^ Much can be learned from other HCP groups but research is needed to address issues specific to the various dental working environments, especially in the context of an increasingly feminised workforce.^52^ Dental-specific research is needed to inform how dentist and DCP victims/survivors can access safety, be heard and supported, recover and continue practising safely, and how colleagues can support those affected. Research must be conducted safely, sensitively and collaboratively. Additionally, research should explore how best to address DA perpetration when an abuser and survivor are employed by the same organisation. All published research should include information for accessing support as described by the Women's Aid research framework.^26^

## Supplementary Information

Supplementary Information (DOCX 29KB)

## References

[1] Scottish Women's Aid. What is domestic abuse? 2024. Available at https://womensaid.scot/information-support/what-is-domestic-abuse/ (accessed 1 November 2025).

[2] Safer Scotland. Domestic abuse. Available at https://safer.scot/information/da/ (accessed 1 November 2025).

[3] UK Parliament. Domestic Abuse Act 2021. 2021. Availalbe at https://www.legislation.gov.uk/ukpga/2021/17/contents/enacted (accessed 1 November 2025).

[4] Scottish Parliament. Domestic Abuse (Scotland) Act 2018. 2018. Available at https://www.legislation.gov.uk/asp/2018/5/contents (accessed 1 November 2025).

[5] Northern Ireland Assembly. Domestic Abuse and Civil Proceedings Act (Northern Ireland) 2021. 2021. Available at https://www.legislation.gov.uk/nia/2021/2 (accessed 1 November 2025).

[6] Brooks-Hay O, Burman M, McFeely C. *Domestic Abuse: Contemporary Perspectives and Innovative Practices*. Edinburgh: Dunedin Academic Press, 2018.

[7] Office for National Statistics. Domestic abuse in England and Wales overview: November 2024. 2024. Available at https://www.ons.gov.uk/peoplepopulationandcommunity/crimeandjustice/bulletins/domesticabuseinenglandandwalesoverview/november2024 (accessed 1 November 2025).

[8] SafeLives. Facts and figures. Available at https://safelives.org.uk/policy-evidence/about-domestic-abuse/how-widespread-domestic-abuse-and-what-impact (accessed 1 November 2025).

[9] Women's Aid. Why do we say domestic abuse is gendered? Available at https://www.womensaid.org.uk/information-support/what-is-domestic-abuse/domestic-abuse-is-a-gendered-crime/ (accessed 1 November 2025).

[10] Council of Europe. Council of Europe Convention on preventing and combating violence against women and domestic violence (CETS No. 210). 2011. Available at https://www.coe.int/en/web/conventions/full-list?module=treaty-detail&treatynum=210 (accessed 1 November 2025).

[11] Women's Aid. Domestic abuse, the facts. Available at https://womensaid.org.uk/what-we-do/research/domestic-abuse-the-facts (accessed 1 November 2025).

[12] Equality and Human Rights Commission. Government review of employment rights for survivors of domestic abuse. 2020. Available at https://www.equalityhumanrights.com/sites/default/files/government_review_of_employment_rights_for_survivors_of_domestic_abuse.pdf (accessed 1 November 2025).

[13] Trades Union Congress. Domestic Violence and the Workplace: a TUC Survey Report. 2014. Available at https://www.tuc.org.uk/research-analysis/reports/domestic-violence-and-workplace (accessed 1 November 2025).

[14] UK Government. The economic and social costs of domestic abuse. 2019. Available at https://www.gov.uk/government/publications/the-economic-and-social-costs-of-domestic-abuse (accessed 1 November 2025).

[15] Crown Prosection Service. Domestic abuse. 2022. Available at https://www.cps.gov.uk/prosecution-guidance/domestic-abuse (accessed 1 November 2025).

[16] McLindon E, Humphreys C, Hegarty K. ‘It happens to clinicians too': an Australian prevalence study of intimate partner and family violence against health professionals. *BMC Womens Health* 2018; **18:** 113. 10.1186/s12905-018-0588-y PMC6020247 29940948

[17] Cavell Nurses' Trust. Skint, shaken yet still caring: but who is caring for our nurses? 2016. Available at https://cavell.org.uk/wp-content/uploads/2019/06/Skint-shaken-yet-still-caring-Key-Findings-Cavell-Nurses-Trust-Final.pdf (accessed 1 November 2025).

[18] Dheensa S, McLindon E, Spencer C *et al*. Healthcare professionals' own experiences of domestic violence and abuse: a meta-analysis of prevalence and systematic review of risk markers and consequences. *Trauma Violence Abuse* 2023; **24:** 1282–1299. 10.1177/15248380211061771 PMC10240650 34978481

[19] O'Doherty L, Hegarty K, Ramsay J, Davidson L L, Feder G, Taft A. Screening women for intimate partner violence in healthcare settings. *Cochrane Database Syst Rev* 2015; DOI: 10.1002/14651858.CD007007.pub3. 10.1002/14651858.CD007007.pub2 23633338

[20] Coulthard P, Hutchison I, Bell J A, Coulthard I D, Kennedy H. COVID-19, domestic violence and abuse, and urgent dental and oral and maxillofacial surgery care. *Br Dent J* 2020; **228:** 923–926. 10.1038/s41415-020-1709-1 PMC7319221 32591703

[21] Coulthard P, Feder G, Evans M *et al*. Dentistry responding to domestic violence and abuse: a dental, practice-based intervention and a feasibility study for a cluster randomised trial. *Br Dent J* 2022; **233:** 949–955. 10.1038/s41415-022-5271-x PMC9734034 36494544

[22] Doughty J L, Ferns H, Taylor-Weetman K *et al*. Dental public health in action: delivering a domestic violence and abuse screening and identification training programme in North Staffordshire-based dental practices. *Community Dent Health* 2023; **40:** 3–8. 10.1922/CDH_00153Doughty06 36696477

[23] Le B T, Dierks E J, Ueeck B A, Homer L D, Potter B F. Maxillofacial injuries associated with domestic violence. *J Oral Maxillofac Surg* 2001; **59:** 1277–1283. 10.1053/joms.2001.27490 11688025

[24] Saddki N, Suhaimi A A, Daud R. Maxillofacial injuries associated with intimate partner violence in women. *BMC Public Health* 2010; **10:** 268. 10.1186/1471-2458-10-268 PMC2882351 20492720

[25] Grant M J, Booth A. A typology of reviews: an analysis of 14 review types and associated methodologies. *Health Inf Libr J* 2009; **26:** 91–108. 10.1111/j.1471-1842.2009.00848.x 19490148

[26] Women's Aid. Research integrity framework on domestic violence and abuse. 2020. Available at https://www.womensaid.org.uk/wp-content/uploads/2020/11/Research-Integrity-Framework-RIF-on-Domestic-Violence-and-Abuse-DVA-November-2020.pdf (accessed 1 November 2025).

[27] Ayiku L, Levay P, Hudson T *et al*. The Embase UK filter: validation of a geographic search filter to retrieve research about the UK from OVID Embase. *Health Inf Libr J* 2019; **36:** 121–133. 10.1111/hir.12252 30912233

[28] Page M J, McKenzie J E, Bossuyt P M *et al*. The PRISMA 2020 statement: an updated guideline for reporting systematic reviews. *BMJ* 2021; DOI: 10.1136/bmj.n71. 10.1136/bmj.n71 PMC8005924 33782057

[29] Dheensa S, Doughty J, Gregory A. Healthcare professionals as domestic abuse survivors: workplace impact and support-seeking. *Occup Med (Lond)* 2024; **74:** 514–522. 10.1093/occmed/kqae070 PMC11444377 39167918

[30] Women's Aid. Why haven't they told me? 2025. Available at https://womensaid.org.uk/information-support/friends-and-family/why-havent-they-told-me/ (accessed 1 November 2025).

[31] Crown Prosecution Service. Domestic abuse: context and challenges. 2025. Available at https://www.cps.gov.uk/domestic-abuse-context-and-challenges (accessed 1 November 2025).

[32] Heron R L, Eisma M C. Barriers and facilitators of disclosing domestic violence to the healthcare service: a systematic review of qualitative research. *Health Soc Care Community* 2021; **29:** 612–630. 10.1111/hsc.13282 PMC8248429 33440034

[33] McGregor K, Stephen-Lewis D, Richards C M, Gilchrist F, Taylor-Dunn H, Jones R. An exploration of healthcare professionals' personal and professional experiences of domestic violence and abuse. 2016. Available at https://research.brighton.ac.uk/files/22350298/An_Exploration_of_Healthcare_Professionals_Personal_and_Professional_experiences_of_Domestic_Violence_and_Abuse_FINAL_.pdf (accessed 1 November 2025).

[34] Royal College of Midwives. Safe places? Workplace support for those experiencing domestic abuse. 2018. Available at https://pureportal.coventry.ac.uk/files/21474534/Domestic_Abuse_report_final.pdf (accessed 1 November 2025).

[35] British Medical Association. Domestic abuse in the health profession report. 2024. Available at https://www.bma.org.uk/advice-and-support/nhs-delivery-and-workforce/creating-a-healthy-workplace/domestic-abuse-in-the-health-profession-report (accessed 1 November 2025).

[36] Donovan E, Santer M, Morgan S, Daker-White G, Willcox M. Domestic abuse among female doctors: thematic analysis of qualitative interviews in the UK. *Br J Gen Pract* 2021; DOI: 10.3399/bjgp.2020.0795. 10.3399/BJGP.2020.0795 PMC7909912 33558329

[37] Myhill A, Hohl K. The ‘golden thread': coercive control and risk assessment for domestic violence. *J Interpers Violence* 2019; **34:** 4477–4497. 10.1177/0886260516675464 27807208

[38] Tarzia L, Cameron J, Watson J *et al*. Personal barriers to addressing intimate partner abuse: a qualitative meta-synthesis of healthcare practitioners' experiences. *BMC Health Serv Res* 2021; **21:** 567. 10.1186/s12913-021-06582-2 PMC8191204 34107941

[39] Hudspeth N, Cameron J, Baloch S, Tarzia L, Hegarty K. Health practitioners' perceptions of structural barriers to the identification of intimate partner abuse: a qualitative meta-synthesis. *BMC Health Serv Res* 2022; **22:** 96. 10.1186/s12913-022-07491-8 PMC8783157 35065630

[40] Bradbury-Jones C, Taylor J, Kroll T, Duncan F. Domestic abuse awareness and recognition among primary healthcare professionals and abused women: a qualitative investigation. *J Clin Nurs* 2014; **23:** 3057–3068. 10.1111/jocn.12534 24444430

[41] Employers' Initiative on Domestic Abuse. The EIDA Handbook. Available at https://www.eida.org.uk/resources/eida-handbook (accessed 1 November 2025).

[42] McGarry J. Domestic violence and abuse: an exploration and evaluation of a domestic abuse nurse specialist role in acute health care services. *J Clin Nurs* 2017; **26:** 2266–2273. 10.1111/jocn.13203 27075361

[43] UK Legislation. Health and Safety at Work etc Act 1974. 1974. Available at https://www.legislation.gov.uk/ukpga/1974/37/contents (accessed 1 November 2025).

[44] UK Legislation. The Management of Health and Safety at Work Regulations 1992. 1992. Available at https://www.legislation.gov.uk/uksi/1992/2051/contents/made (accesed 1 November 2025). 10.1177/146642409711700112 9050295

[45] UK Legisltation. The Reporting of Injuries, Diseases and Dangerous Occurrences Regulations 2013. 2013. Available at https://www.legislation.gov.uk/uksi/2013/1471/contents (accessed 1 November 2025).

[46] UK Legisltation. The Health and Safety (Consultation with Employees) Regulations 1996. 1996. Available at https://www.legislation.gov.uk/uksi/1996/1513 (accessed 1 November 2025).

[47] UK Government. Tackling domestic abuse plan. 2022. Available at https://www.gov.uk/government/publications/tackling-domestic-abuse-plan (accessed 1 November 2025).

[48] UK Government. Workplace support for victims of domestic abuse. 2021. Available at https://assets.publishing.service.gov.uk/media/60005da1e90e07639c70cf69/workplace-support-for-victims-of-domestic-abuse-report.pdf (accessed 1 November 2025).

[49] The Chartered Institute of Personnel and Development. Domestic abuse: guidance for people professionals on managing and supporting employees. 2023. Available at https://www.cipd.org/uk/knowledge/guides/supporting-employees-experiencing-domestic-abuse/ (accessed 1 November 2025).

[50] Giesbrecht C J. The impact of intimate partner violence in the workplace: results of a saskatchewan survey. *J Interpers Violence* 2022; **37:** 1960–1973. 10.1177/0886260520921875 32468907

[51] Wibberley G, Bennett T, Jones C, Hollinrake A. The role of trade unions in supporting victims of domestic violence in the workplace. *Indust Relat J* 2018; **49:** 69–85.

[52] General Dental Council. Registration statistical report 2023. 2023. Available at https://www.gdc-uk.org/docs/default-source/registration-reports/registration-statistical-report-2023---final-and-accessible-v2.pdf?sfvrsn=91957fe_3 (accessed 1 November 2025).

