# Supplementary material for: Domestic abuse experienced by healthcare practitioners: what about dentists and dental care professionals? A systematised review
Source: Br Dent J. 2025 Dec 12;239(11):768–73. doi: 10.1038/s41415-025-9011-x (PMC12700814; doi:10.1038/s41415-025-9011-x)
Supplement: Supplementary file 1 — Supplementary Information (DOCX 29KB) [file 41415_2025_9011_MOESM1_ESM.docx]

**Source: CINAHL**

Interface: EBSCOhost

Database coverage dates: 1981 to date

Search date: 21 August 2023

Retrieved records: 198

Search Strategy:

S1 (MH "Dentists+") 11,079

S2 (MH "Dentistry+") 108,564

S3 TI ( (dental or dentist*) ) OR AB ( (dental or dentist*) ) 81,390

S4 (MH "Dental Auxiliaries+") 11,450

S5 TI ( (((dental or dentist*) n1 auxiliar*) or "dental therapist#" or "oral health therapist#") ) OR AB ( (((dental or dentist*) n1 auxiliar*) or "dental therapist#" or "oral health therapist#") ) 409

S6 TI ( (denturist# or "clinical dent* technician#" or "maxillofacial technician#") ) OR AB ( (denturist# or "clinical dent* technician#" or "maxillofacial technician#") ) 18

S7 TI ( (orthodonti* n1 (auxiliary or auxiliaries or therapist#)) ) OR AB ( (orthodonti* n1 (auxiliary or auxiliaries or therapist#)) ) 13

S8 TI ( ((denturist# or endodonti* or maxillofacial or orthodont*) n1 (assistant# or hygienist# or technician# or technologist#)) ) OR AB ( ((denturist# or endodonti* or maxillofacial or orthodont*) n1 (assistant# or hygienist# or technician# or technologist#)) ) 19

S9 TI ( (("oral health*" or "oral surger*" or "oral care") n1 (hygienist# or assistant# or technician# or nurse# or therapist# or auxiliar* or personnel or practitioner* or student*)) ) OR AB ( (("oral health*" or "oral surger*" or "oral care") n1 (hygienist# or assistant# or technician# or nurse# or therapist# or auxiliar* or personnel or practitioner* or student*)) ) 425

S10 TI "oral hygienist#" OR AB "oral hygienist#" 17

S11 TI ( ("oral surg*" n1 (assistant# or hygienist#)) ) OR AB ( ("oral surg*" n1 (assistant# or hygienist#)) ) 3

S12 TI ( ((chairside or "chair-side") n2 (hygienist# or technician# or nurse# or dentistry or assistant# or assistance or assisting or auxiliar* or personnel# or professional# or employee# or staff# or worker#)) ) OR AB ( ((chairside or "chair-side") n2 (hygienist# or technician# or nurse# or dentistry or assistant# or assistance or assisting or auxiliar* or personnel# or professional# or employee# or staff# or worker#)) ) 41

S13 TI ( ("four hand* dent*" or "six hand* dent*") ) OR AB ( ("four hand* dent*" or "six hand* dent*") ) 4

S14 TI ( (hygienist# n3 (advanc* or expand* or extend* or increase* or role#)) ) OR AB ( (hygienist# n3 (advanc* or expand* or extend* or increase* or role#)) ) 404

S15 S1 OR S2 OR S3 OR S4 OR S5 OR S6 OR S7 OR S8 OR S9 OR S10 OR S11 OR S12 OR S13 OR S14 156,831

S16 (MH "Domestic Violence") 10,452

S17 (MH "Intimate Partner Violence") 12,728

S18 (MH "Battered Women") 4,307

S19 TI ( (control* n2 (partner# or domestic or spous* or husband# or wife or wives or wom?n or mother* or coercive or famil* or household or relation* or marital or dating or conjugal or hono#r)) ) OR AB ( (control* n2 (partner# or domestic or spous* or husband# or wife or wives or wom?n or mother* or coercive or famil* or household or relation* or marital or dating or conjugal or hono#r)) ) 16,797

S20 (MH "Gender-Based Violence") 947

S21 (MH "Sexual Abuse+") 20,644

S22 TI ( ((abus* or violen* or victim* or harass*) n2 (partner# or domestic or spous* or husband# or wife or wives or wom?n or mother* or coercive or famil* or household or relation* or marital or dating or gender* or conjugal or hono#r or emotional* or psychological* or physical* or sex*)) ) OR AB ( ((abus* or violen* or victim* or harass*) n2 (partner# or domestic or spous* or husband# or wife or wives or wom?n or mother* or coercive or famil* or household or relation* or marital or dating or gender* or conjugal or hono#r or emotional* or psychological* or physical* or sex*)) ) 47,824

S23 TI ( ((batter* or beat*) n2 (partner# or domestic or spous* or husband# or wife or wives or wom?n or mother* or coercive or famil* or household or relation* or marital or dating or gender* or conjugal or hono#r)) ) OR AB ( ((batter* or beat*) n2 (partner# or domestic or spous* or husband# or wife or wives or wom?n or mother* or coercive or famil* or household or relation* or marital or dating or gender* or conjugal or hono#r)) ) 1,147

S24 TI ( (rape* or sex* offen#es) ) OR AB ( (rape* or sex* offen#es) ) 3,887

S25 TI ( (forced n (prostitution or abortion or sterili?ation)) ) OR AB ( (forced n (prostitution or abortion or sterili?ation)) ) 1

S26 (MH "Female Genital Mutilation") 1,661

S27 TI ( ("Female Genital Mutilation" or FGM or "female circumcision" or clitorectomy or clitoridectomy or infibulation) ) OR AB ( ("Female Genital Mutilation" or FGM or "female circumcision" or clitorectomy or clitoridectomy or infibulation) ) 1,451

S28 TI ( ((hono#r n2 kill*) or femicide or (female* and homicide)) ) OR AB ( ((hono#r n2 kill*) or femicide or (female* and homicide)) ) 580

S29 (MH "Homicide") NOT (MH "Female") 3,511

S30 (MH "Survivors") NOT ((MH "Neoplasms+") OR TI (HNC or cancer*) OR AB (HNC or cancer*)) 10,422

S31 (TI survivor* OR AB survivor*) NOT ((MH "Neoplasms+") OR TI (HNC or cancer*) OR AB (HNC or cancer*)) 32,056

S32 TI ( (((lived or own or personal) n experience#) or PWLE) ) OR AB ( (((lived or own or personal) n experience#) or PWLE) ) 120

S33 S16 OR S17 OR S18 OR S19 OR S20 OR S21 OR S22 OR S23 OR S24 OR S25 OR S26 OR S27 OR S28 OR S29 OR S30 OR S31 OR S32 114,131

S34 (MH "United Kingdom+") 321,473

S35 TI ( ("national health service*" or nhs*) ) OR AB ( ("national health service*" or nhs*) ) 43,584

S36 TI ( (english not ((published or publication* or translat* or written or language* or speak* or literature or citation*) n4 english)) ) OR AB ( (english not ((published or publication* or translat* or written or language* or speak* or literature or citation*) n4 english)) ) 21,717

S37 TI ( (gb or "g.b." or britain* or (british* not "british columbia") or uk or "u.k." or "united kingdom*" or (england* not "new england") or "northern ireland*" or "northern irish*" or scotland* or scottish* or ((wales or "south wales") not "new south wales") or welsh*) ) OR AB ( (gb or "g.b." or britain* or (british* not "british columbia") or uk or "u.k." or "united kingdom*" or (england* not "new england") or "northern ireland*" or "northern irish*" or scotland* or scottish* or ((wales or "south wales") not "new south wales") or welsh*) ) OR SO ( (gb or "g.b." or britain* or (british* not "british columbia") or uk or "u.k." or "united kingdom*" or (england* not "new england") or "northern ireland*" or "northern irish*" or scotland* or scottish* or ((wales or "south wales") not "new south wales") or welsh*) ) OR AF ( (gb or "g.b." or britain* or (british* not "british columbia") or uk or "u.k." or "united kingdom*" or (england* not "new england") or "northern ireland*" or "northern irish*" or scotland* or scottish* or ((wales or "south wales") not "new south wales") or welsh*) ) 738,143

S38 TI ( (bath or "bath's" or ((birmingham not alabama*) or ("birmingham's" not alabama*) or bradford or "bradford's" or brighton or "brighton's" or bristol or "bristol's" or carlisle* or "carlisle's" or (cambridge not (massachusetts* or boston* or harvard*)) or ("cambridge's" not (massachusetts* or boston* or harvard*)) or (canterbury not zealand*) or ("canterbury's" not zealand*) or chelmsford or "chelmsford's" or chester or "chester's" or chichester or "chichester's" or coventry or "coventry's" or derby or "derby's" or (durham not (carolina* or nc)) or ("durham's" not (carolina* or nc)) or ely or "ely's" or exeter or "exeter's" or gloucester or "gloucester's" or hereford or "hereford's" or hull or "hull's" or lancaster or "lancaster's" or leeds* or leicester or "leicester's" or (lincoln not nebraska*) or ("lincoln's" not nebraska*) or (liverpool not (new south wales* or nsw)) or ("liverpool's" not ("new south wales*" or nsw)) or ((london not (ontario* or ont or toronto*)) or ("london's" not (ontario* or ont or toronto*)) or manchester or "manchester's" or (newcastle not ("new south wales*" or nsw)) or ("newcastle's" not ("new south wales*" or nsw)) or norwich or "norwich's" or nottingham or "nottingham's" or oxford or "oxford's" or peterborough or "peterborough's" or plymouth or "plymouth's" or portsmouth or "portsmouth's" or preston or "preston's" or ripon or "ripon's" or salford or "salford's" or salisbury or "salisbury's" or sheffield or "sheffield's" or southampton or "southampton's" or "st albans" or stoke or "stoke's" or sunderland or "sunderland's" or truro or "truro's" or wakefield or "wakefield's" or wells or westminster or "westminster's" or winchester or "winchester's" or wolverhampton or "wolverhampton's" or (worcester not (massachusetts* or boston* or harvard*)) or ("worcester's" not (massachusetts* or boston* or harvard*)) or (york not ("new york*" or ny or ontario* or ont or toronto*)) or ("york's" not ("new york*" or ny or ontario* or ont or toronto*))))) ) OR AB ( (bath or "bath's" or ((birmingham not alabama*) or ("birmingham's" not alabama*) or bradford or "bradford's" or brighton or "brighton's" or bristol or "bristol's" or carlisle* or "carlisle's" or (cambridge not (massachusetts* or boston* or harvard*)) or ("cambridge's" not (massachusetts* or boston* or harvard*)) or (canterbury not zealand*) or ("canterbury's" not zealand*) or chelmsford or "chelmsford's" or chester or "chester's" or chichester or "chichester's" or coventry or "coventry's" or derby or "derby's" or (durham not (carolina* or nc)) or ("durham's" not (carolina* or nc)) or ely or "ely's" or exeter or "exeter's" or gloucester or "gloucester's" or hereford or "hereford's" or hull or "hull's" or lancaster or "lancaster's" or leeds* or leicester or "leicester's" or (lincoln not nebraska*) or ("lincoln's" not nebraska*) or (liverpool not (new south wales* or nsw)) or ("liverpool's" not ("new south wales*" or nsw)) or ((london not (ontario* or ont or toronto*)) or ("london's" not (ontario* or ont or toronto*)) or manchester or "manchester's" or (newcastle not ("new south wales*" or nsw)) or ("newcastle's" not ("new south wales*" or nsw)) or norwich or "norwich's" or nottingham or "nottingham's" or oxford or "oxford's" or peterborough or "peterborough's" or plymouth or "plymouth's" or portsmouth or "portsmouth's" or preston or "preston's" or ripon or "ripon's" or salford or "salford's" or salisbury or "salisbury's" or sheffield or "sheffield's" or southampton or "southampton's" or "st albans" or stoke or "stoke's" or sunderland or "sunderland's" or truro or "truro's" or wakefield or "wakefield's" or wells or westminster or "westminster's" or winchester or "winchester's" or wolverhampton or "wolverhampton's" or (worcester not (massachusetts* or boston* or harvard*)) or ("worcester's" not (massachusetts* or boston* or harvard*)) or (york not ("new york*" or ny or ontario* or ont or toronto*)) or ("york's" not ("new york*" or ny or ontario* or ont or toronto*))))) ) OR AF ( (bath or "bath's" or ((birmingham not alabama*) or ("birmingham's" not alabama*) or bradford or "bradford's" or brighton or "brighton's" or bristol or "bristol's" or carlisle* or "carlisle's" or (cambridge not (massachusetts* or boston* or harvard*)) or ("cambridge's" not (massachusetts* or boston* or harvard*)) or (canterbury not zealand*) or ("canterbury's" not zealand*) or chelmsford or "chelmsford's" or chester or "chester's" or chichester or "chichester's" or coventry or "coventry's" or derby or "derby's" or (durham not (carolina* or nc)) or ("durham's" not (carolina* or nc)) or ely or "ely's" or exeter or "exeter's" or gloucester or "gloucester's" or hereford or "hereford's" or hull or "hull's" or lancaster or "lancaster's" or leeds* or leicester or "leicester's" or (lincoln not nebraska*) or ("lincoln's" not nebraska*) or (liverpool not (new south wales* or nsw)) or ("liverpool's" not ("new south wales*" or nsw)) or ((london not (ontario* or ont or toronto*)) or ("london's" not (ontario* or ont or toronto*)) or manchester or "manchester's" or (newcastle not ("new south wales*" or nsw)) or ("newcastle's" not ("new south wales*" or nsw)) or norwich or "norwich's" or nottingham or "nottingham's" or oxford or "oxford's" or peterborough or "peterborough's" or plymouth or "plymouth's" or portsmouth or "portsmouth's" or preston or "preston's" or ripon or "ripon's" or salford or "salford's" or salisbury or "salisbury's" or sheffield or "sheffield's" or southampton or "southampton's" or "st albans" or stoke or "stoke's" or sunderland or "sunderland's" or truro or "truro's" or wakefield or "wakefield's" or wells or westminster or "westminster's" or winchester or "winchester's" or wolverhampton or "wolverhampton's" or (worcester not (massachusetts* or boston* or harvard*)) or ("worcester's" not (massachusetts* or boston* or harvard*)) or (york not ("new york*" or ny or ontario* or ont or toronto*)) or ("york's" not ("new york*" or ny or ontario* or ont or toronto*))))) ) 1,098,896

S39 TI ( (bangor or "bangor's" or cardiff or "cardiff's" or newport or "newport's" or "st asaph" or "st asaph's" or "st davids" or swansea or "swansea's") ) OR AB ( (bangor or "bangor's" or cardiff or "cardiff's" or newport or "newport's" or "st asaph" or "st asaph's" or "st davids" or swansea or "swansea's") ) OR AF ( (bangor or "bangor's" or cardiff or "cardiff's" or newport or "newport's" or "st asaph" or "st asaph's" or "st davids" or swansea or "swansea's") ) 25,413

S40 TI ( (aberdeen or "aberdeen's" or dundee or "dundee's" or edinburgh or "edinburgh's" or glasgow or "glasgow's" or inverness or (perth not australia*) or ("perth's" not australia*) or stirling or "stirling's") ) OR AB ( (aberdeen or "aberdeen's" or dundee or "dundee's" or edinburgh or "edinburgh's" or glasgow or "glasgow's" or inverness or (perth not australia*) or ("perth's" not australia*) or stirling or "stirling's") ) OR AF ( (aberdeen or "aberdeen's" or dundee or "dundee's" or edinburgh or "edinburgh's" or glasgow or "glasgow's" or inverness or (perth not australia*) or ("perth's" not australia*) or stirling or "stirling's") ) 71,111

S41 TI ( (armagh or "armagh's" or belfast or "belfast's" or lisburn or "lisburn's" or londonderry or "londonderry's" or derry or "derry's" or newry or "newry's") ) OR AB ( (armagh or "armagh's" or belfast or "belfast's" or lisburn or "lisburn's" or londonderry or "londonderry's" or derry or "derry's" or newry or "newry's") ) OR AF ( (armagh or "armagh's" or belfast or "belfast's" or lisburn or "lisburn's" or londonderry or "londonderry's" or derry or "derry's" or newry or "newry's") ) 11,258

S42 S34 OR S35 OR S36 OR S37 OR S38 OR S39 OR S40 OR S41 1,571,779

S43 ((MH "Africa") OR (MH "America") OR (MH "Antarctic Regions") OR (MH "Arctic Regions") OR (MH "Asia") OR (MH "Atlantic Islands") OR (MH "Australia") OR (MH "Indian Ocean Islands") OR (MH "Pacific Islands")) NOT ((MH "United Kingdom+") OR (MH "Europe")) 97,699

S44 S42 NOT S43 1,556,278

S45 S15 AND S33 AND S44 199

S46 S15 AND S33 AND S44 Limiters - English Language (198)

**Source: Embase 1947-Present, updated daily**

Interface: OvidSP

Database coverage dates: 1947 to present

Search date: 21 August 2023

Retrieved records: 224

Search Strategy:

1  exp dentistry/ (141651)
2  dental practice/ (2266)
3  (dental or dentist*).tw. (323471)
4  exp dental auxiliary/ (13984)
5  exp dental personnel/ (44241)
6  (((dental or dentist*) adj2 auxiliar*) or dental therapist? or oral health therapist?).tw. (1186)
7  (denturist? or clinical dent* technician? or maxillofacial technician?).tw. (126)
8  (orthodonti* adj2 (auxiliary or auxiliaries or therapist?)).tw. (68)
9  ((denturist? or endodonti* or maxillofacial or orthodont*) adj2 (assistant? or hygienist? or technician? or technologist?)).tw. (62)
10  ((oral health* or oral surger* or oral care) adj2 (hygienist? or assistant? or technician? or nurse? or therapist? or auxiliar* or personnel or practitioner* or student*)).tw. (672)
11  oral hygienist?.tw. (92)
12  (oral surg* adj2 (assistant? or hygienist?)).tw. (7)
13  ((chairside or chair-side) adj3 (hygienist? or technician? or nurse? or dentistry or assistant? or assistance or assisting or auxiliar* or personnel? or professional? or employee? or staff? or worker?)).tw. (142)
14  (four hand* dent* or six hand* dent*).tw. (56)
15  (hygienist? adj4 (advanc* or expand* or extend* or increase* or role?)).tw. (423)
16  or/1-15 (423849)
17  domestic violence/ (11621)
18  exp partner violence/ (16920)
19  family violence/ (4187)
20  battered woman/ (3342)
21  (control* adj3 (partner? or domestic or spous* or husband? or wife or wives or wom#n or mother* or coercive or famil* or household or relation* or marital or dating or conjugal or hono?r)).tw. (71788)
22  gender based violence/ (1581)
23  sexual crime/ (13807)
24  ((abus* or violen* or victim* or harass*) adj3 (partner? or domestic or spous* or husband? or wife or wives or wom#n or mother* or coercive or famil* or household or relation* or marital or dating or gender* or conjugal or hono?r or emotional* or psychological* or physical* or sex*)).tw. (82089)
25  ((batter* or beat*) adj3 (partner? or domestic or spous* or husband? or wife or wives or wom#n or mother* or coercive or famil* or household or relation* or marital or dating or gender* or conjugal or hono?r)).tw. (2359)
26  (rape* or sex* offen?es).tw. (17138)
27  (forced adj (prostitution or abortion or sterili#ation)).tw. (146)
28  exp female genital mutilation/ (1968)
29  (Female Genital Mutilation or FGM or female circumcision or clitorectomy or clitoridectomy or infibulation).tw. (3401)
30  ((hono?r adj3 kill*) or femicide or (female* and homicide)).tw. (1485)
31  homicide/ and female/ (7206)
32  survivor/ not (exp neoplasm/ or (hnc or cancer*).tw.) (49870)
33  survivor*.tw. not (exp neoplasm/ or (hnc or scoping).tw.) (125746)
34  (((lived or own or personal) adj experience?) or PWLE).tw. (51743)
35  or/17-34 (366055)
36  exp United Kingdom/ (496143)
37  (national health service* or nhs*).ti,ab,in,ad. (469740)
38  (english not ((published or publication* or translat* or written or language* or speak* or literature or citation*) adj5 english)).ti,ab. (62213)
39  (gb or "g.b." or britain* or (british* not "british columbia") or uk or "u.k." or united kingdom* or (england* not "new england") or northern ireland* or northern irish* or scotland* or scottish* or ((wales or "south wales") not "new south wales") or welsh*).ti,ab,jw,in. (3790139)
40  (bath or "bath's" or ((birmingham not alabama*) or ("birmingham's" not alabama*) or bradford or "bradford's" or brighton or "brighton's" or bristol or "bristol's" or carlisle* or "carlisle's" or (cambridge not (massachusetts* or boston* or harvard*)) or ("cambridge's" not (massachusetts* or boston* or harvard*)) or (canterbury not zealand*) or ("canterbury's" not zealand*) or chelmsford or "chelmsford's" or chester or "chester's" or chichester or "chichester's" or coventry or "coventry's" or derby or "derby's" or (durham not (carolina* or nc)) or ("durham's" not (carolina* or nc)) or ely or "ely's" or exeter or "exeter's" or gloucester or "gloucester's" or hereford or "hereford's" or hull or "hull's" or lancaster or "lancaster's" or leeds* or leicester or "leicester's" or (lincoln not nebraska*) or ("lincoln's" not nebraska*) or (liverpool not (new south wales* or nsw)) or ("liverpool's" not (new south wales* or nsw)) or ((london not (ontario* or ont or toronto*)) or ("london's" not (ontario* or ont or toronto*)) or manchester or "manchester's" or (newcastle not (new south wales* or nsw)) or ("newcastle's" not (new south wales* or nsw)) or norwich or "norwich's" or nottingham or "nottingham's" or oxford or "oxford's" or peterborough or "peterborough's" or plymouth or "plymouth's" or portsmouth or "portsmouth's" or preston or "preston's" or ripon or "ripon's" or salford or "salford's" or salisbury or "salisbury's" or sheffield or "sheffield's" or southampton or "southampton's" or st albans or stoke or "stoke's" or sunderland or "sunderland's" or truro or "truro's" or wakefield or "wakefield's" or wells or westminster or "westminster's" or winchester or "winchester's" or wolverhampton or "wolverhampton's" or (worcester not (massachusetts* or boston* or harvard*)) or ("worcester's" not (massachusetts* or boston* or harvard*)) or (york not ("new york*" or ny or ontario* or ont or toronto*)) or ("york's" not ("new york*" or ny or ontario* or ont or toronto*))))).ti,ab,in. (3001979)
41  (bangor or "bangor's" or cardiff or "cardiff's" or newport or "newport's" or st asaph or "st asaph's" or st davids or swansea or "swansea's").ti,ab,in. (121702)
42  (aberdeen or "aberdeen's" or dundee or "dundee's" or edinburgh or "edinburgh's" or glasgow or "glasgow's" or inverness or (perth not australia*) or ("perth's" not australia*) or stirling or "stirling's").ti,ab,in. (415384)
43  (armagh or "armagh's" or belfast or "belfast's" or lisburn or "lisburn's" or londonderry or "londonderry's" or derry or "derry's" or newry or "newry's").ti,ab,in. (57879)
44  or/36-43 (4712464)
45  (exp africa/ or exp americas/ or exp antarctic regions/ or exp arctic regions/ or exp asia/ or exp australia/ or exp oceania/) not (exp United Kingdom/ or europe/) (3818497)
46  44 not 45 (4446830)
47  16 and 35 and 46 (226)
48  limit 47 to english language (224)

**Source: MEDLINE ALL**

Interface: OvidSP

Database coverage dates: 1946 to 18 August 2023

Search date: 21 August 2023

Retrieved records: 263

Search Strategy:

1  exp Dentists/ (20990)
2  exp Dentistry/ (434364)
3  Practice Management, Dental/ (11215)
4  (dental or dentist*).tw. (300917)
5  exp Dental Auxiliaries/ (13473)
6  exp Dental Staff/ (2481)
7  (((dental or dentist*) adj2 auxiliar*) or dental therapist? or oral health therapist?).tw. (1148)
8  (denturist? or clinical dent* technician? or maxillofacial technician?).tw. (125)
9  (orthodonti* adj2 (auxiliary or auxiliaries or therapist?)).tw. (61)
10  ((denturist? or endodonti* or maxillofacial or orthodont*) adj2 (assistant? or hygienist? or technician? or technologist?)).tw. (57)
11  ((oral health* or oral surger* or oral care) adj2 (hygienist? or assistant? or technician? or nurse? or therapist? or auxiliar* or personnel or practitioner* or student*)).tw. (636)
12  oral hygienist?.tw. (84)
13  (oral surg* adj2 (assistant? or hygienist?)).tw. (6)
14  ((chairside or chair-side) adj3 (hygienist? or technician? or nurse? or dentistry or assistant? or assistance or assisting or auxiliar* or personnel? or professional? or employee? or staff? or worker?)).tw. (129)
15  (four hand* dent* or six hand* dent*).tw. (48)
16  (hygienist? adj4 (advanc* or expand* or extend* or increase* or role?)).tw. (414)
17  or/1-16 (601260)
18  domestic violence/ or spouse abuse/ (14574)
19  Battered Women/ (2717)
20  exp Intimate Partner Violence/ (13276)
21  (control* adj3 (partner? or domestic or spous* or husband? or wife or wives or wom#n or mother* or coercive or famil* or household or relation* or marital or dating or conjugal or hono?r)).tw. (52961)
22  Gender-Based Violence/ (591)
23  exp Sex Offenses/ (27679)
24  ((abus* or violen* or victim* or harass*) adj3 (partner? or domestic or spous* or husband? or wife or wives or wom#n or mother* or coercive or famil* or household or relation* or marital or dating or gender* or conjugal or hono?r or emotional* or psychological* or physical* or sex*)).tw. (67655)
25  ((batter* or beat*) adj3 (partner? or domestic or spous* or husband? or wife or wives or wom#n or mother* or coercive or famil* or household or relation* or marital or dating or gender* or conjugal or hono?r)).tw. (1937)
26  (rape* or sex* offen?es).tw. (15405)
27  (forced adj (prostitution or abortion or sterili#ation)).tw. (127)
28  Circumcision, Female/ (1708)
29  (Female Genital Mutilation or FGM or female circumcision or clitorectomy or clitoridectomy or infibulation).tw. (2764)
30  ((hono?r adj3 kill*) or femicide or (female* and homicide)).tw. (1175)
31  Homicide/ and Female/ (6053)
32  Survivors/ not (exp Neoplasms/ or (HNC or cancer*).tw.) (15305)
33  survivor*.tw. not (exp Neoplasms/ or (HNC or cancer*).tw.) (83220)
34  (((lived or own or personal) adj experience?) or PWLE).tw. (35735)
35  or/18-34 (269830)
36  exp United Kingdom/ (390683)
37  (national health service* or nhs*).ti,ab,in. (272870)
38  (english not ((published or publication* or translat* or written or language* or speak* or literature or citation*) adj5 english)).ti,ab. (50085)
39  (gb or "g.b." or britain* or (british* not "british columbia") or uk or "u.k." or united kingdom* or (england* not "new england") or northern ireland* or northern irish* or scotland* or scottish* or ((wales or "south wales") not "new south wales") or welsh*).ti,ab,jw,in. (2460167)
40  (bath or "bath's" or ((birmingham not alabama*) or ("birmingham's" not alabama*) or bradford or "bradford's" or brighton or "brighton's" or bristol or "bristol's" or carlisle* or "carlisle's" or (cambridge not (massachusetts* or boston* or harvard*)) or ("cambridge's" not (massachusetts* or boston* or harvard*)) or (canterbury not zealand*) or ("canterbury's" not zealand*) or chelmsford or "chelmsford's" or chester or "chester's" or chichester or "chichester's" or coventry or "coventry's" or derby or "derby's" or (durham not (carolina* or nc)) or ("durham's" not (carolina* or nc)) or ely or "ely's" or exeter or "exeter's" or gloucester or "gloucester's" or hereford or "hereford's" or hull or "hull's" or lancaster or "lancaster's" or leeds* or leicester or "leicester's" or (lincoln not nebraska*) or ("lincoln's" not nebraska*) or (liverpool not (new south wales* or nsw)) or ("liverpool's" not (new south wales* or nsw)) or ((london not (ontario* or ont or toronto*)) or ("london's" not (ontario* or ont or toronto*)) or manchester or "manchester's" or (newcastle not (new south wales* or nsw)) or ("newcastle's" not (new south wales* or nsw)) or norwich or "norwich's" or nottingham or "nottingham's" or oxford or "oxford's" or peterborough or "peterborough's" or plymouth or "plymouth's" or portsmouth or "portsmouth's" or preston or "preston's" or ripon or "ripon's" or salford or "salford's" or salisbury or "salisbury's" or sheffield or "sheffield's" or southampton or "southampton's" or st albans or stoke or "stoke's" or sunderland or "sunderland's" or truro or "truro's" or wakefield or "wakefield's" or wells or westminster or "westminster's" or winchester or "winchester's" or wolverhampton or "wolverhampton's" or (worcester not (massachusetts* or boston* or harvard*)) or ("worcester's" not (massachusetts* or boston* or harvard*)) or (york not ("new york*" or ny or ontario* or ont or toronto*)) or ("york's" not ("new york*" or ny or ontario* or ont or toronto*))))).ti,ab,in. (1755838)
41  (aberdeen or "aberdeen's" or dundee or "dundee's" or edinburgh or "edinburgh's" or glasgow or "glasgow's" or inverness or (perth not australia*) or ("perth's" not australia*) or stirling or "stirling's").ti,ab,in. (258583)
42  (armagh or "armagh's" or belfast or "belfast's" or lisburn or "lisburn's" or londonderry or "londonderry's" or derry or "derry's" or newry or "newry's").ti,ab,in. (34035)
43  or/36-42 (3080682)
44  (exp africa/ or exp americas/ or exp antarctic regions/ or exp arctic regions/ or exp asia/ or exp australia/ or exp oceania/) not (exp United Kingdom/ or europe/) (3339706)
45  43 not 44 (2916246)
46  17 and 35 and 45 (267)
47  limit 46 to english language (263)

**Source: CINAHL**

Interface: EBSCOhost

Database coverage dates: 1981 to date

Search date: 13 September 2024

Retrieved records: 7

Search Strategy:

S1 (MH "Dentists+") 11,560

S2 (MH "Dentistry+") 112,909

S3 TI ( (dental or dentist*) ) OR AB ( (dental or dentist*) ) 78,547

S4 (MH "Dental Auxiliaries+") 11,857

S5 TI ( (((dental or dentist*) n1 auxiliar*) or "dental therapist#" or "oral health therapist#") ) OR AB ( (((dental or dentist*) n1 auxiliar*) or "dental therapist#" or "oral health therapist#") ) 394

S6 TI ( (denturist# or "clinical dent* technician#" or "maxillofacial technician#") ) OR AB ( (denturist# or "clinical dent* technician#" or "maxillofacial technician#") ) 18

S7 TI ( (orthodonti* n1 (auxiliary or auxiliaries or therapist#)) ) OR AB ( (orthodonti* n1 (auxiliary or auxiliaries or therapist#)) ) 13

S8 TI ( ((denturist# or endodonti* or maxillofacial or orthodont*) n1 (assistant# or hygienist# or technician# or technologist#)) ) OR AB ( ((denturist# or endodonti* or maxillofacial or orthodont*) n1 (assistant# or hygienist# or technician# or technologist#)) ) 16

S9 TI ( (("oral health*" or "oral surger*" or "oral care") n1 (hygienist# or assistant# or technician# or nurse# or therapist# or auxiliar* or personnel or practitioner* or student*)) ) OR AB ( (("oral health*" or "oral surger*" or "oral care") n1 (hygienist# or assistant# or technician# or nurse# or therapist# or auxiliar* or personnel or practitioner* or student*)) ) 445

S10 TI "oral hygienist#" OR AB "oral hygienist#" 15

S11 TI ( ("oral surg*" n1 (assistant# or hygienist#)) ) OR AB ( ("oral surg*" n1 (assistant# or hygienist#)) ) 3

S12 TI ( ((chairside or "chair-side") n2 (hygienist# or technician# or nurse# or dentistry or assistant# or assistance or assisting or auxiliar* or personnel# or professional# or employee# or staff# or worker#)) ) OR AB ( ((chairside or "chair-side") n2 (hygienist# or technician# or nurse# or dentistry or assistant# or assistance or assisting or auxiliar* or personnel# or professional# or employee# or staff# or worker#)) ) 36

S13 TI ( ("four hand* dent*" or "six hand* dent*") ) OR AB ( ("four hand* dent*" or "six hand* dent*") ) 3

S14 TI ( (hygienist# n3 (advanc* or expand* or extend* or increase* or role#)) ) OR AB ( (hygienist# n3 (advanc* or expand* or extend* or increase* or role#)) ) 290

S15 S1 OR S2 OR S3 OR S4 OR S5 OR S6 OR S7 OR S8 OR S9 OR S10 OR S11 OR S12 OR S13 OR S14 161,033

S16 (MH "Domestic Violence") 9,874

S17 (MH "Intimate Partner Violence") 11,004

S18 (MH "Battered Women") 0

S19 TI ( (control* n2 (partner# or domestic or spous* or husband# or wife or wives or wom?n or mother* or coercive or famil* or household or relation* or marital or dating or conjugal or hono#r)) ) OR AB ( (control* n2 (partner# or domestic or spous* or husband# or wife or wives or wom?n or mother* or coercive or famil* or household or relation* or marital or dating or conjugal or hono#r)) ) 16,919

S20 (MH "Gender-Based Violence") 1,006

S21 (MH "Sexual Abuse+") 19,943

S22 TI ( ((abus* or violen* or victim* or harass*) n2 (partner# or domestic or spous* or husband# or wife or wives or wom?n or mother* or coercive or famil* or household or relation* or marital or dating or gender* or conjugal or hono#r or emotional* or psychological* or physical* or sex*)) ) OR AB ( ((abus* or violen* or victim* or harass*) n2 (partner# or domestic or spous* or husband# or wife or wives or wom?n or mother* or coercive or famil* or household or relation* or marital or dating or gender* or conjugal or hono#r or emotional* or psychological* or physical* or sex*)) ) 44,833

S23 TI ( ((batter* or beat*) n2 (partner# or domestic or spous* or husband# or wife or wives or wom?n or mother* or coercive or famil* or household or relation* or marital or dating or gender* or conjugal or hono#r)) ) OR AB ( ((batter* or beat*) n2 (partner# or domestic or spous* or husband# or wife or wives or wom?n or mother* or coercive or famil* or household or relation* or marital or dating or gender* or conjugal or hono#r)) ) 985

S24 TI ( (rape* or sex* offen#es) ) OR AB ( (rape* or sex* offen#es) ) 3,551

S25 TI ( (forced n (prostitution or abortion or sterili?ation)) ) OR AB ( (forced n (prostitution or abortion or sterili?ation)) ) 0

S26 (MH "Female Genital Mutilation") 1,695

S27 TI ( ("Female Genital Mutilation" or FGM or "female circumcision" or clitorectomy or clitoridectomy or infibulation) ) OR AB ( ("Female Genital Mutilation" or FGM or "female circumcision" or clitorectomy or clitoridectomy or infibulation) ) 1,416

S28 TI ( ((hono#r n2 kill*) or femicide or (female* and homicide)) ) OR AB ( ((hono#r n2 kill*) or femicide or (female* and homicide)) ) 541

S29 (MH "Homicide") NOT (MH "Female") 3,536

S30 (MH "Survivors") NOT ((MH "Neoplasms+") OR TI (HNC or cancer*) OR AB (HNC or cancer*)) 10,629

S31 (TI survivor* OR AB survivor*) NOT ((MH "Neoplasms+") OR TI (HNC or cancer*) OR AB (HNC or cancer*)) 32,484

S32 TI ( (((lived or own or personal) n experience#) or PWLE) ) OR AB ( (((lived or own or personal) n experience#) or PWLE) ) 151

S33 S16 OR S17 OR S18 OR S19 OR S20 OR S21 OR S22 OR S23 OR S24 OR S25 OR S26 OR S27 OR S28 OR S29 OR S30 OR S31 OR S32 112,069

S34 (MH "United Kingdom+") 324,902

S35 TI ( ("national health service*" or nhs*) ) OR AB ( ("national health service*" or nhs*) ) 38,730

S36 TI ( (english not ((published or publication* or translat* or written or language* or speak* or literature or citation*) n4 english)) ) OR AB ( (english not ((published or publication* or translat* or written or language* or speak* or literature or citation*) n4 english)) ) 22,486

S37 TI ( (gb or "g.b." or britain* or (british* not "british columbia") or uk or "u.k." or "united kingdom*" or (england* not "new england") or "northern ireland*" or "northern irish*" or scotland* or scottish* or ((wales or "south wales") not "new south wales") or welsh*) ) OR AB ( (gb or "g.b." or britain* or (british* not "british columbia") or uk or "u.k." or "united kingdom*" or (england* not "new england") or "northern ireland*" or "northern irish*" or scotland* or scottish* or ((wales or "south wales") not "new south wales") or welsh*) ) OR SO ( (gb or "g.b." or britain* or (british* not "british columbia") or uk or "u.k." or "united kingdom*" or (england* not "new england") or "northern ireland*" or "northern irish*" or scotland* or scottish* or ((wales or "south wales") not "new south wales") or welsh*) ) OR AF ( (gb or "g.b." or britain* or (british* not "british columbia") or uk or "u.k." or "united kingdom*" or (england* not "new england") or "northern ireland*" or "northern irish*" or scotland* or scottish* or ((wales or "south wales") not "new south wales") or welsh*) ) 743,932

S38 TI ( (bath or "bath's" or ((birmingham not alabama*) or ("birmingham's" not alabama*) or bradford or "bradford's" or brighton or "brighton's" or bristol or "bristol's" or carlisle* or "carlisle's" or (cambridge not (massachusetts* or boston* or harvard*)) or ("cambridge's" not (massachusetts* or boston* or harvard*)) or (canterbury not zealand*) or ("canterbury's" not zealand*) or chelmsford or "chelmsford's" or chester or "chester's" or chichester or "chichester's" or coventry or "coventry's" or derby or "derby's" or (durham not (carolina* or nc)) or ("durham's" not (carolina* or nc)) or ely or "ely's" or exeter or "exeter's" or gloucester or "gloucester's" or hereford or "hereford's" or hull or "hull's" or lancaster or "lancaster's" or leeds* or leicester or "leicester's" or (lincoln not nebraska*) or ("lincoln's" not nebraska*) or (liverpool not (new south wales* or nsw)) or ("liverpool's" not ("new south wales*" or nsw)) or ((london not (ontario* or ont or toronto*)) or ("london's" not (ontario* or ont or toronto*)) or manchester or "manchester's" or (newcastle not ("new south wales*" or nsw)) or ("newcastle's" not ("new south wales*" or nsw)) or norwich or "norwich's" or nottingham or "nottingham's" or oxford or "oxford's" or peterborough or "peterborough's" or plymouth or "plymouth's" or portsmouth or "portsmouth's" or preston or "preston's" or ripon or "ripon's" or salford or "salford's" or salisbury or "salisbury's" or sheffield or "sheffield's" or southampton or "southampton's" or "st albans" or stoke or "stoke's" or sunderland or "sunderland's" or truro or "truro's" or wakefield or "wakefield's" or wells or westminster or "westminster's" or winchester or "winchester's" or wolverhampton or "wolverhampton's" or (worcester not (massachusetts* or boston* or harvard*)) or ("worcester's" not (massachusetts* or boston* or harvard*)) or (york not ("new york*" or ny or ontario* or ont or toronto*)) or ("york's" not ("new york*" or ny or ontario* or ont or toronto*))))) ) OR AB ( (bath or "bath's" or ((birmingham not alabama*) or ("birmingham's" not alabama*) or bradford or "bradford's" or brighton or "brighton's" or bristol or "bristol's" or carlisle* or "carlisle's" or (cambridge not (massachusetts* or boston* or harvard*)) or ("cambridge's" not (massachusetts* or boston* or harvard*)) or (canterbury not zealand*) or ("canterbury's" not zealand*) or chelmsford or "chelmsford's" or chester or "chester's" or chichester or "chichester's" or coventry or "coventry's" or derby or "derby's" or (durham not (carolina* or nc)) or ("durham's" not (carolina* or nc)) or ely or "ely's" or exeter or "exeter's" or gloucester or "gloucester's" or hereford or "hereford's" or hull or "hull's" or lancaster or "lancaster's" or leeds* or leicester or "leicester's" or (lincoln not nebraska*) or ("lincoln's" not nebraska*) or (liverpool not (new south wales* or nsw)) or ("liverpool's" not ("new south wales*" or nsw)) or ((london not (ontario* or ont or toronto*)) or ("london's" not (ontario* or ont or toronto*)) or manchester or "manchester's" or (newcastle not ("new south wales*" or nsw)) or ("newcastle's" not ("new south wales*" or nsw)) or norwich or "norwich's" or nottingham or "nottingham's" or oxford or "oxford's" or peterborough or "peterborough's" or plymouth or "plymouth's" or portsmouth or "portsmouth's" or preston or "preston's" or ripon or "ripon's" or salford or "salford's" or salisbury or "salisbury's" or sheffield or "sheffield's" or southampton or "southampton's" or "st albans" or stoke or "stoke's" or sunderland or "sunderland's" or truro or "truro's" or wakefield or "wakefield's" or wells or westminster or "westminster's" or winchester or "winchester's" or wolverhampton or "wolverhampton's" or (worcester not (massachusetts* or boston* or harvard*)) or ("worcester's" not (massachusetts* or boston* or harvard*)) or (york not ("new york*" or ny or ontario* or ont or toronto*)) or ("york's" not ("new york*" or ny or ontario* or ont or toronto*))))) ) OR AF ( (bath or "bath's" or ((birmingham not alabama*) or ("birmingham's" not alabama*) or bradford or "bradford's" or brighton or "brighton's" or bristol or "bristol's" or carlisle* or "carlisle's" or (cambridge not (massachusetts* or boston* or harvard*)) or ("cambridge's" not (massachusetts* or boston* or harvard*)) or (canterbury not zealand*) or ("canterbury's" not zealand*) or chelmsford or "chelmsford's" or chester or "chester's" or chichester or "chichester's" or coventry or "coventry's" or derby or "derby's" or (durham not (carolina* or nc)) or ("durham's" not (carolina* or nc)) or ely or "ely's" or exeter or "exeter's" or gloucester or "gloucester's" or hereford or "hereford's" or hull or "hull's" or lancaster or "lancaster's" or leeds* or leicester or "leicester's" or (lincoln not nebraska*) or ("lincoln's" not nebraska*) or (liverpool not (new south wales* or nsw)) or ("liverpool's" not ("new south wales*" or nsw)) or ((london not (ontario* or ont or toronto*)) or ("london's" not (ontario* or ont or toronto*)) or manchester or "manchester's" or (newcastle not ("new south wales*" or nsw)) or ("newcastle's" not ("new south wales*" or nsw)) or norwich or "norwich's" or nottingham or "nottingham's" or oxford or "oxford's" or peterborough or "peterborough's" or plymouth or "plymouth's" or portsmouth or "portsmouth's" or preston or "preston's" or ripon or "ripon's" or salford or "salford's" or salisbury or "salisbury's" or sheffield or "sheffield's" or southampton or "southampton's" or "st albans" or stoke or "stoke's" or sunderland or "sunderland's" or truro or "truro's" or wakefield or "wakefield's" or wells or westminster or "westminster's" or winchester or "winchester's" or wolverhampton or "wolverhampton's" or (worcester not (massachusetts* or boston* or harvard*)) or ("worcester's" not (massachusetts* or boston* or harvard*)) or (york not ("new york*" or ny or ontario* or ont or toronto*)) or ("york's" not ("new york*" or ny or ontario* or ont or toronto*))))) ) 1,118,266

S39 TI ( (bangor or "bangor's" or cardiff or "cardiff's" or newport or "newport's" or "st asaph" or "st asaph's" or "st davids" or swansea or "swansea's") ) OR AB ( (bangor or "bangor's" or cardiff or "cardiff's" or newport or "newport's" or "st asaph" or "st asaph's" or "st davids" or swansea or "swansea's") ) OR AF ( (bangor or "bangor's" or cardiff or "cardiff's" or newport or "newport's" or "st asaph" or "st asaph's" or "st davids" or swansea or "swansea's") ) 26,360

S40 TI ( (aberdeen or "aberdeen's" or dundee or "dundee's" or edinburgh or "edinburgh's" or glasgow or "glasgow's" or inverness or (perth not australia*) or ("perth's" not australia*) or stirling or "stirling's") ) OR AB ( (aberdeen or "aberdeen's" or dundee or "dundee's" or edinburgh or "edinburgh's" or glasgow or "glasgow's" or inverness or (perth not australia*) or ("perth's" not australia*) or stirling or "stirling's") ) OR AF ( (aberdeen or "aberdeen's" or dundee or "dundee's" or edinburgh or "edinburgh's" or glasgow or "glasgow's" or inverness or (perth not australia*) or ("perth's" not australia*) or stirling or "stirling's") ) 73,519

S41 TI ( (armagh or "armagh's" or belfast or "belfast's" or lisburn or "lisburn's" or londonderry or "londonderry's" or derry or "derry's" or newry or "newry's") ) OR AB ( (armagh or "armagh's" or belfast or "belfast's" or lisburn or "lisburn's" or londonderry or "londonderry's" or derry or "derry's" or newry or "newry's") ) OR AF ( (armagh or "armagh's" or belfast or "belfast's" or lisburn or "lisburn's" or londonderry or "londonderry's" or derry or "derry's" or newry or "newry's") ) 11,798

S42 S34 OR S35 OR S36 OR S37 OR S38 OR S39 OR S40 OR S41 1,594,046

S43 ((MH "Africa") OR (MH "America") OR (MH "Antarctic Regions") OR (MH "Arctic Regions") OR (MH "Asia") OR (MH "Atlantic Islands") OR (MH "Australia") OR (MH "Indian Ocean Islands") OR (MH "Pacific Islands")) NOT ((MH "United Kingdom+") OR (MH "Europe")) 99,152

S44 S42 NOT S43 1,578,366

S45 S15 AND S33 AND S44 203

S46 S15 AND S33 AND S44 Limiters - English Language 201

S47 S15 AND S33 AND S44 Limiters - Publication Date: 20230801-; English Language 7

**Source: Embase 1947-Present, updated daily**

Interface: OvidSP

Database coverage dates: 1947 to present

Search date: 13 September 2024

Retrieved records: 19

Search Strategy:

1 exp dentistry/ 144497

2 dental practice/ 2612

3 (dental or dentist*).tw. 339477

4 exp dental auxiliary/ 14242

5 exp dental personnel/ 45879

6 (((dental or dentist*) adj2 auxiliar*) or dental therapist? or oral health therapist?).tw. 1224

7 (denturist? or clinical dent* technician? or maxillofacial technician?).tw. 127

8 (orthodonti* adj2 (auxiliary or auxiliaries or therapist?)).tw. 71

9 ((denturist? or endodonti* or maxillofacial or orthodont*) adj2 (assistant? or hygienist? or technician? or technologist?)).tw. 66

10 ((oral health* or oral surger* or oral care) adj2 (hygienist? or assistant? or technician? or nurse? or therapist? or auxiliar* or personnel or practitioner* or student*)).tw. 729

11 oral hygienist?.tw. 94

12 (oral surg* adj2 (assistant? or hygienist?)).tw. 8

13 ((chairside or chair-side) adj3 (hygienist? or technician? or nurse? or dentistry or assistant? or assistance or assisting or auxiliar* or personnel? or professional? or employee? or staff? or worker?)).tw. 145

14 (four hand* dent* or six hand* dent*).tw. 56

15 (hygienist? adj4 (advanc* or expand* or extend* or increase* or role?)).tw. 436

16 or/1-15 441122

17 domestic violence/ 12510

18 exp partner violence/ 18337

19 family violence/ 4332

20 battered woman/ 3371

21 (control* adj3 (partner? or domestic or spous* or husband? or wife or wives or wom#n or mother* or coercive or famil* or household or relation* or marital or dating or conjugal or hono?r)).tw. 75163

22 gender based violence/ 1901

23 sexual crime/ 14369

24 ((abus* or violen* or victim* or harass*) adj3 (partner? or domestic or spous* or husband? or wife or wives or wom#n or mother* or coercive or famil* or household or relation* or marital or dating or gender* or conjugal or hono?r or emotional* or psychological* or physical* or sex*)).tw. 87852

25 ((batter* or beat*) adj3 (partner? or domestic or spous* or husband? or wife or wives or wom#n or mother* or coercive or famil* or household or relation* or marital or dating or gender* or conjugal or hono?r)).tw. 2463

26 (rape* or sex* offen?es).tw. 18050

27 (forced adj (prostitution or abortion or sterili#ation)).tw. 153

28 exp female genital mutilation/ 2120

29 (Female Genital Mutilation or FGM or female circumcision or clitorectomy or clitoridectomy or infibulation).tw. 3677

30 ((hono?r adj3 kill*) or femicide or (female* and homicide)).tw. 1578

31 homicide/ and female/ 7648

32 survivor/ not (exp neoplasm/ or (hnc or cancer*).tw.) 52721

33 survivor*.tw. not (exp neoplasm/ or (hnc or scoping).tw.) 134595

34 (((lived or own or personal) adj experience?) or PWLE).tw. 56356

35 or/17-34 390274

36 exp United Kingdom/ 514152

37 (national health service* or nhs*).ti,ab,in,ad. 511015

38 (english not ((published or publication* or translat* or written or language* or speak* or literature or citation*) adj5 english)).ti,ab. 68579

39 (gb or "g.b." or britain* or (british* not "british columbia") or uk or "u.k." or united kingdom* or (england* not "new england") or northern ireland* or northern irish* or scotland* or scottish* or ((wales or "south wales") not "new south wales") or welsh*).ti,ab,jw,in. 3978942

40 (bath or "bath's" or ((birmingham not alabama*) or ("birmingham's" not alabama*) or bradford or "bradford's" or brighton or "brighton's" or bristol or "bristol's" or carlisle* or "carlisle's" or (cambridge not (massachusetts* or boston* or harvard*)) or ("cambridge's" not (massachusetts* or boston* or harvard*)) or (canterbury not zealand*) or ("canterbury's" not zealand*) or chelmsford or "chelmsford's" or chester or "chester's" or chichester or "chichester's" or coventry or "coventry's" or derby or "derby's" or (durham not (carolina* or nc)) or ("durham's" not (carolina* or nc)) or ely or "ely's" or exeter or "exeter's" or gloucester or "gloucester's" or hereford or "hereford's" or hull or "hull's" or lancaster or "lancaster's" or leeds* or leicester or "leicester's" or (lincoln not nebraska*) or ("lincoln's" not nebraska*) or (liverpool not (new south wales* or nsw)) or ("liverpool's" not (new south wales* or nsw)) or ((london not (ontario* or ont or toronto*)) or ("london's" not (ontario* or ont or toronto*)) or manchester or "manchester's" or (newcastle not (new south wales* or nsw)) or ("newcastle's" not (new south wales* or nsw)) or norwich or "norwich's" or nottingham or "nottingham's" or oxford or "oxford's" or peterborough or "peterborough's" or plymouth or "plymouth's" or portsmouth or "portsmouth's" or preston or "preston's" or ripon or "ripon's" or salford or "salford's" or salisbury or "salisbury's" or sheffield or "sheffield's" or southampton or "southampton's" or st albans or stoke or "stoke's" or sunderland or "sunderland's" or truro or "truro's" or wakefield or "wakefield's" or wells or westminster or "westminster's" or winchester or "winchester's" or wolverhampton or "wolverhampton's" or (worcester not (massachusetts* or boston* or harvard*)) or ("worcester's" not (massachusetts* or boston* or harvard*)) or (york not ("new york*" or ny or ontario* or ont or toronto*)) or ("york's" not ("new york*" or ny or ontario* or ont or toronto*))))).ti,ab,in. 3161995

41 (bangor or "bangor's" or cardiff or "cardiff's" or newport or "newport's" or st asaph or "st asaph's" or st davids or swansea or "swansea's").ti,ab,in. 128562

42 (aberdeen or "aberdeen's" or dundee or "dundee's" or edinburgh or "edinburgh's" or glasgow or "glasgow's" or inverness or (perth not australia*) or ("perth's" not australia*) or stirling or "stirling's").ti,ab,in. 437360

43 (armagh or "armagh's" or belfast or "belfast's" or lisburn or "lisburn's" or londonderry or "londonderry's" or derry or "derry's" or newry or "newry's").ti,ab,in. 61388

44 or/36-43 4947253

45 (exp africa/ or exp americas/ or exp antarctic regions/ or exp arctic regions/ or exp asia/ or exp australia/ or exp oceania/) not (exp United Kingdom/ or europe/) 4023127

46 44 not 45 4661410

47 16 and 35 and 46 246

48 limit 47 to english language 244

49 limit 48 to dc=20230822-20240913 19

**Source: MEDLINE ALL**

Interface: OvidSP

Database coverage dates: 1946 to 12 September 2024

Search date: 13 September 2024

Retrieved records: 17

Search Strategy:

1 exp Dentists/ 21519

2 exp Dentistry/ 444192

3 Practice Management, Dental/ 11221

4 (dental or dentist*).tw. 317632

5 exp Dental Auxiliaries/ 13577

6 exp Dental Staff/ 34947

7 (((dental or dentist*) adj2 auxiliar*) or dental therapist? or oral health therapist?).tw. 1187

8 (denturist? or clinical dent* technician? or maxillofacial technician?).tw. 127

9 (orthodonti* adj2 (auxiliary or auxiliaries or therapist?)).tw. 63

10 ((denturist? or endodonti* or maxillofacial or orthodont*) adj2 (assistant? or hygienist? or technician? or technologist?)).tw. 60

11 ((oral health* or oral surger* or oral care) adj2 (hygienist? or assistant? or technician? or nurse? or therapist? or auxiliar* or personnel or practitioner* or student*)).tw. 701

12 oral hygienist?.tw. 86

13 (oral surg* adj2 (assistant? or hygienist?)).tw. 7

14 ((chairside or chair-side) adj3 (hygienist? or technician? or nurse? or dentistry or assistant? or assistance or assisting or auxiliar* or personnel? or professional? or employee? or staff? or worker?)).tw. 133

15 (four hand* dent* or six hand* dent*).tw. 49

16 (hygienist? adj4 (advanc* or expand* or extend* or increase* or role?)).tw. 427

17 or/1-16 623503

18 domestic violence/ or spouse abuse/ 14865

19 Battered Women/ 2726

20 exp Intimate Partner Violence/ 14083

21 (control* adj3 (partner? or domestic or spous* or husband? or wife or wives or wom#n or mother* or coercive or famil* or household or relation* or marital or dating or conjugal or hono?r)).tw. 55292

22 Gender-Based Violence/ 724

23 exp Sex Offenses/ 28726

24 ((abus* or violen* or victim* or harass*) adj3 (partner? or domestic or spous* or husband? or wife or wives or wom#n or mother* or coercive or famil* or household or relation* or marital or dating or gender* or conjugal or hono?r or emotional* or psychological* or physical* or sex*)).tw. 72566

25 ((batter* or beat*) adj3 (partner? or domestic or spous* or husband? or wife or wives or wom#n or mother* or coercive or famil* or household or relation* or marital or dating or gender* or conjugal or hono?r)).tw. 2014

26 (rape* or sex* offen?es).tw. 16362

27 (forced adj (prostitution or abortion or sterili#ation)).tw. 134

28 Circumcision, Female/ 1766

29 (Female Genital Mutilation or FGM or female circumcision or clitorectomy or clitoridectomy or infibulation).tw. 2985

30 ((hono?r adj3 kill*) or femicide or (female* and homicide)).tw. 1258

31 Homicide/ and Female/ 6214

32 Survivors/ not (exp Neoplasms/ or (HNC or cancer*).tw.) 16166

33 survivor*.tw. not (exp Neoplasms/ or (HNC or cancer*).tw.) 88911

34 (((lived or own or personal) adj experience?) or PWLE).tw. 39571

35 or/18-34 287227

36 exp United Kingdom/ 398304

37 (national health service* or nhs*).ti,ab,in. 298189

38 (english not ((published or publication* or translat* or written or language* or speak* or literature or citation*) adj5 english)).ti,ab. 132834

39 (gb or "g.b." or britain* or (british* not &quot;british columbia&quot;) or uk or "u.k." or united kingdom* or (england* not "new england") or northern ireland* or northern irish* or scotland* or scottish* or ((wales or "south wales") not "new south wales") or welsh*).ti,ab,jw,in. 2589100

40 (bath or "bath's" or ((birmingham not alabama*) or ("birmingham's" not alabama*) or bradford or "bradford's" or brighton or "brighton's" or bristol or "bristol's" or carlisle* or "carlisle's" or (cambridge not (massachusetts* or boston* or harvard*)) or ("cambridge's" not (massachusetts* or boston* or harvard*)) or (canterbury not zealand*) or ("canterbury's" not zealand*) or chelmsford or "chelmsford's" or chester or "chester's" or chichester or "chichester's" or coventry or "coventry's" or derby or "derby's" or (durham not (carolina* or nc)) or ("durham's" not (carolina* or nc)) or ely or "ely's" or exeter or "exeter's" or gloucester or "gloucester's" or hereford or "hereford's" or hull or "hull's" or lancaster or "lancaster's" or leeds* or leicester or "leicester's" or (lincoln not nebraska*) or ("lincoln's" not nebraska*) or (liverpool not (new south wales* or nsw)) or ("liverpool's" not (new south wales* or nsw)) or ((london not (ontario* or ont or toronto*)) or ("london's" not (ontario* or ont or toronto*)) or manchester or "manchester's" or (newcastle not (new south wales* or nsw)) or ("newcastle's" not (new south wales* or nsw)) or norwich or "norwich's" or nottingham or "nottingham's" or oxford or "oxford's" or peterborough or "peterborough's" or plymouth or "plymouth's" or portsmouth or "portsmouth's" or preston or "preston's" or ripon or "ripon's" or salford or "salford's" or salisbury or "salisbury's" or sheffield or "sheffield's" or southampton or "southampton's" or st albans or stoke or "stoke's" or sunderland or "sunderland's" or truro or "truro's" or wakefield or "wakefield's" or wells or westminster or "westminster's" or winchester or "winchester's" or wolverhampton or "wolverhampton's" or (worcester not (massachusetts* or boston* or harvard*)) or ("worcester's" not (massachusetts* or boston* or harvard*)) or (york not ("new york*" or ny or ontario* or ont or toronto*)) or ("york's" not ("new york*" or ny or ontario* or ont or toronto*))))).ti,ab,in. 1871137

41 (aberdeen or "aberdeen's" or dundee or "dundee's" or edinburgh or "edinburgh's" or glasgow or "glasgow's" or inverness or (perth not australia*) or ("perth's" not australia*) or stirling or "stirling's").ti,ab,in. 275226

42 (armagh or "armagh's" or belfast or "belfast's" or lisburn or "lisburn's" or londonderry or "londonderry's" or derry or "derry's" or newry or "newry's").ti,ab,in. 36684

43 or/36-42 3312665

44 (exp africa/ or exp americas/ or exp antarctic regions/ or exp arctic regions/ or exp asia/ or exp australia/ or exp oceania/) not (exp United Kingdom/ or europe/) 3466946

45 43 not 44 3105840

46 17 and 35 and 45 287

47 limit 46 to english language 282

48 ("20230822" or "20230823" or "20230824" or "20230825" or "20230825" or "20230826" or "20230827" or "20230828" or "20230829" or "2023083*" or "202309*" or "20231*" or "2024*").dt,ez,ed. 1845660

49 47 and 48 17
